# Salvage surgery for local failures after stereotactic ablative radiotherapy for early stage non-small cell lung cancer

**DOI:** 10.1186/s13014-016-0706-7

**Published:** 2016-10-03

**Authors:** Naomi E. Verstegen, Alexander P. W. M. Maat, Frank J. Lagerwaard, Marinus A. Paul, Michel I Versteegh, Joris J. Joosten, Willem Lastdrager, Egbert F. Smit, Ben J. Slotman, Joost J. M. E. Nuyttens, Suresh Senan

**Affiliations:** 1 Department of Radiation Oncology, VU University Medical Center Amsterdam, De Boelelaan 1117, Postbox 7057, 1007 MB Amsterdam, The Netherlands; 2 Department of Cardiothoracic Surgery, Erasmus Medical Center Rotterdam, Rotterdam, The Netherlands; 3 Department of Cardiothoracic Surgery, VU University Medical Center Amsterdam, Amsterdam, The Netherlands; 4 Department of Cardiothoracic Surgery, Leiden University Medical Center, Leiden, The Netherlands; 5 Department of Surgery, Westfriesgasthuis Hoorn, Hoorn, The Netherlands; 6 Department of Surgery, Gelre Hospital Apeldoorn, Apeldoorn, The Netherlands; 7 Department of Thoracic Oncology, Netherlands Cancer Institute, Amsterdam, The Netherlands; 8 Department of Radiation Oncology, Erasmus Medical Center Rotterdam, Rotterdam, The Netherlands

## Abstract

**Introduction:**

The literature on surgical salvage, i.e. lung resections in patients who develop a local recurrence following stereotactic ablative radiotherapy (SABR), is limited. We describe our experience with salvage surgery in nine patients who developed a local recurrence following SABR for early stage non-small cell lung cancer (NSCLC).

**Methods:**

Patients who underwent surgical salvage for a local recurrence following SABR for NSCLC were identified from two Dutch institutional databases. Complications were scored using the Dindo-Clavien-classification.

**Results:**

Nine patients who underwent surgery for a local recurrence were identified. Median time to local recurrence was 22 months. Recurrences were diagnosed with CT- and/or 18FDG-PET-imaging, with four patients also having a pre-surgical pathological diagnosis. Extensive adhesions were observed during two resections, requiring conversion from a thoracoscopic procedure to thoracotomy during one of these procedures. Three patients experienced complications post-surgery; grade 2 (*N* = 2) and grade 3a (*N* = 1), respectively. All resection specimens showed viable tumor cells. Median length of hospital stay was 8 days (range 5–15 days) and 30-day mortality was 0 %. Lymph node dissection revealed mediastinal metastases in 3 patients, all of whom received adjuvant therapy.

**Conclusions:**

Our experience with nine surgical procedures for local recurrences post-SABR revealed two grade IIIa complications, and a 30-day mortality of 0 %, suggesting that salvage surgery can be safely performed after SABR.

## Introduction

Surgery is the standard of care for operable patients with early stage non-small cell lung cancer (NSCLC). Stereotactic ablative radiotherapy (SABR) is the recommended therapy in patients unfit for surgery, as well as for patients who decline surgery [1, 2]. Approximately 10 % of patients who undergo SABR to guideline-recommended doses will develop local tumor recurrences [3]. A growing number of reports have addressed salvage surgery following SABR for early stage lung cancer [4 – 9]. All but one of the published reports on surgical salvage post-SABR have originated from Japanese centers, with one possibly being an update of a previous report [4]. This suggests that the available literature represents only between 22–27 patients in total. Encouragingly, the 5-year overall survival (OS) in 12 patients who underwent salvage surgery in the largest reported series to date, was 80 % [5]. As the number of potentially operable patients undergoing SABR continues to increase [6], we analyzed the outcomes of salvage surgery in a cohort of Dutch patients who experienced local failure following SABR for a primary early stage NSCLC.

## Materials & methods

We queried the institutional databases at both the VU University Medical Center (VUmc) and the Erasmus Medical Center Rotterdam (EMCR), which represent two of the earliest adopters of SABR for lung cancer in the Netherlands [7, 8], in order to identify patients who had undergone a surgical resection for a local recurrence following SABR for early stage NSCLC. We also contacted surgeons from other major regional surgical centers to determine if they had operated on post-SABR cases.

The delivery of lung SABR in the Netherlands is performed in accordance with published guidelines [9]. Patients from the VUmc were treated to individualized target volumes encompassing all motion on four-dimensional CT scans, with no active motion management [10]. Patients at the EMCR were treated using a Cyberknife unit with real-time tumor tracking [8]. All fractionation schemes had a biologically effective dose of >100 Gy_10_ to the planning target volume (PTV). For each patient, we retrospectively calculated the maximum dose delivered to the proximal bronchial tree.

Post-SABR follow-up consisted of a contrast-enhanced CT-scan of the thorax and abdomen carried out at 2–3 months after treatment, 6-monthly for 2 years, and annually thereafter. For patients followed up elsewhere, their attending lung physicians and surgeons were contacted for details of local recurrence, and any subsequent surgery. Local failure was defined as a suspected recurrence in, or adjacent to, the PTV. The diagnosis of local recurrence was based on CT and/or ^18^FDG PET scans, and discussed within multi-disciplinary tumor boards. Complications following surgery were classified using the Dindo-Clavien classification [11]. The Dindo-Clavien classification is a commonly used and validated classification system for post-operative complications [12]. Follow-up was calculated using the reverse Kaplan-Meier method [13]. Time-to-event outcomes were analyzed using the Kaplan–Meier method.

## Results

Of 11 potential patients identified for further study, nine patients were finally included in this report. One patient was excluded as details of both the surgical procedure and early post-operative course were unavailable, even though this patient finally died of unrelated causes 39 months after salvage surgery. A second excluded patient had prior large-field conventional thoracic radiotherapy, in addition to SABR. All nine patients had been initially considered to be medically operable at the time of diagnosis (Table 1), but had been referred for SABR following discussions at a multidisciplinary tumor board, and with patients themselves. Six of these patients had undergone their SABR procedure at the VUmc, one at the EMCR, and one each at two other Dutch centers. The median Charlson co-morbidity index [14] at initial presentation was 2 (range 1 - 4). All patients initially presented with peripheral tumors, with a mean diameter of 27 mm (SD ± 3.5 mm), and a median planning target volume (PTV) of 23.9 mm^3^ (range 9.2 – 243 mm^3^). The maximum total dose to the ipsilateral proximal bronchial tree on the SABR plan was below 30 Gy in all patients, and below 20 Gy in all but one patient.

**Table 1:** Patient characteristics

	Patients undergoing salvage surgery (*N* = 9) N (%) or Median (range)
Age (years)	65 (54–71)
Gender	
- Male - Female	6 (67 %) 3 (33 %)
Tumor location	
- Left upper lobe - Left lower lobe - Right upper lobe - Right middle lobe	1 (11 %) 4 (44 % 3 (33 %) 1 (11 %)
Fractionation scheme	
- 3 x 20 Gy - 5 x 11 Gy - 5 x 12 Gy - 8 x 7.5 Gy	2 (22 %) 4 (44 %) 1 (11 %) 2 (22 %)
Diagnosis of LR	
- CT + ^18^FDG-PET + path - CT + ^18^FDG-PET	5 (56 %) 4 (44 %)
Time to local recurrence	22 months (10–35)
Initial tumor stage	
- T1aN0M0 - T1bN0M0 - T2aN0M0 - T2bN0M0	4 (44 %) 2 (22 %) 2 (22 %) 1 (11 %)
Pathology prior to SABR	
- Adenocarcinoma - Squamous cell - NSCLC not specified - Not obtained	2 (22 %) 3 (33 %) 2 (22 %) 2 (22 %)

*LR* local recurrence, *CT* Computed tomography imaging, *PET* Positron emission tomography imaging, *Path* pre-surgical pathology

Median time to diagnosis of local recurrence following SABR, which was determined after discussion at multidisciplinary tumor boards, was 22 months (range 10–35 months). At time of recurrence, all patients had a progressive lesion on consecutive CT-scans, which showed local uptake on ^18^FDG-PET scans. Pulmonary function tests before surgery revealed a mean predicted FEV1% of 71 % ± 21 %. Pre-operative pathology-confirmation of local recurrence was made in 4 patients, with another patient having suspicious endobronchial cytology. As assessed on ^18^FDG-PET scans, seven patients had an isolated local recurrence, one patient had a synchronous solitary adrenal gland metastasis in addition to the local recurrence, and another patient showed a histologically confirmed recurrence in a hilar node.

Details of the surgical procedures performed are summarized in Table 2. Six patients underwent a lobectomy, of which one procedure was a full video-assisted thoracoscopic procedure (VATS). Another planned VATS lobectomy was converted to an open pneumonectomy when extensive peritumoral and pleural adhesions were encountered. In yet another patient, a planned sleeve-lobectomy was performed as a pre-operative hilar lymph node metastasis had been identified. In one patient, an intra-operative decision was made to perform only a wedge-resection as the lesion was considered to be very small.

**Table 2:** Surgical outcome

	N (%) or Median (range)
Type of resection	
- Lobectomy - Sleeve-lobectomy - Pneumonectomy - Wedge resection	6 (67 %) 1 (11 %) 1 (11 %) 1 (11 %))
Intra-operative findings	
- No adhesions - Limited adhesions - Extensive adhesions	4 (44 %) 3 (33 %) 2 (22 %)
pTNM	
- T1N0 - T2N0 - T1N2 - T2N2 - T3N0 - T3N2 - T4N0	2 (22 %) 2 (22 %) 1 (11 %) 1 (11 %) 1 (11 %) 1 (11 %) 1 (11 %)
Radicality of resection	
- R0 - R2	8 (89 %) 1 (11 %)
Surgical complications	
- None - Grade 2 - Grade 3a	6 (67 %) 2 (22 %) 1 (11 %)
Length of hospital stay (days)	8 (5–15)

No unexpected intra-operative findings were observed in four of nine resections. Limited intra-thoracic adhesions were observed in three patients, and extensive adhesions were observed during two procedures. Of the latter, one resulted in conversion into an open pneumonectomy in a patient with extensive peritumoral and pleural adhesions, and tumor invasion into pericardial fat. With the exception of the last case, a complete (R0) resection was obtained in all patients. The bronchial stump was covered with an intercostal muscle flap in just one patient. Three patients experienced grade 2 or higher complications following surgery, which in two cases was due to an infection treated with oral antibiotics (grade II complication). One other patient developed a persistent airway leakage, which required a new thoracic tube (grade IIIa complication). This was not the patient in who the bronchial stump was covered using an intercostal muscle flap. None of the patients developed a broncho-pleural fistel. The median length of hospital stay was 8 days (range 5–15 days) The resected sample revealed the presence of viable tumor in all cases, with co-existing necrosis observed in three patients, and fibrosis in four cases. The 30-day mortality was 0 %. One patient died within 90 days following surgery due to disease progression (90-day mortality 11 %).

Five patients had their disease upstaged during surgery: two had a final diagnosis of a T3 tumor and three had mediastinal lymph node metastases. All upstaged patients underwent adjuvant therapy consisting of either chemotherapy (*n* = 4) or radiotherapy (*n* = 1). Median follow-up after surgery was 19 months, with only two patients developing further disease-progression. Of the latter, one developed regional failure, followed by distant metastases, while the other patient developed distant metastasis. Median overall survival for all patients was 26 months, a figure that should be interpreted with caution given the small size of our patient group and the relatively short period of follow-up.

## Discussion

The main finding of our study in patients who underwent salvage surgery following SABR for a peripheral early-stage NSCLC, is that surgery is feasible with little morbidity. The surgical procedures were generally not complicated by SABR-induced fibrosis, except in one patient in whom co-existing infiltrative tumor recurrence could have contributed to ‘adhesions’. However, it should be noted that all nine patients had been considered fit to undergo surgery at the time of undergoing SABR for their index tumor. Our findings can be placed in context by considering SABR outcomes from the VUMC institutional database, where six of the nine patients had been treated. Of the 855 patients from the VUMC who underwent SABR for a stage I NSCLC, 46 developed a local recurrence, with actuarial local control rates at 3 and 5 years being 92.4 and 90.9 %, respectively [3]. Of these 46 patients, 54 % of local recurrences were isolated events, while 13 % of patients developed both local and regional recurrences. It has to be noted, however, that the majority of SABR patients treated at the VUmc were considered to be medically inoperable, both initially and at the time of disease recurrence. Overall, ten patients underwent radical salvage treatment, including the six patients described who underwent salvage surgery.

All of our patients had received SABR doses which were recommended in the ESMO guidelines, namely that of a minimum biological dose of 100 Gy to the tumor-encompassing isodose [1]. Consequently, peritumoral doses may have been higher than were reported in Japanese patients, who mainly received a prescription of a dose of 48 Gy to the tumor isocenter [4, 5, 15–17]. As such, the low rate of observed post-operative complications in our report are reassuring, and consistent with those reported previously. Surgery may also have been well tolerated due to the fact that all our cases, as well as 9 of 12 reported by Hamaji et al, were considered to be fit to undergo surgery at the time of their initial treatment with SABR [5]. These authors initially attempted a VATS approach in six patients, but conversion to open thoracotomy was needed in one patient because of intraoperative findings [5]. They also covered the bronchial stump with a pericardial fat pad in two patients and intercostal muscle in one. Besides a postoperative air leak exceeding 5 days seen in three patients, no postoperative complications were noted in the Japanese series. In that aspect it is important to realize that all patients in our series had been treated for peripheral lesions, and therefore the radiation dose to the ipsilateral proximal bronchial tree had been relatively low. The results of this study therefore cannot be generalized to more centrally located lesions.

The recent changes in patterns of care for patients with early-stage NSCLC are reflected in the increasing numbers of potentially operable patients being referred for SABR [6, 18]. Even though randomized controlled trials comparing surgery and SABR in operable patients have failed to complete accrual [19], several studies using propensity score matching, matched pair analysis, Markov modeling and meta-analytic methodologies for patients with early stage NSCLC reveal comparable outcomes for both treatment modalities [20, 21]. At present, nearly half of all Dutch pulmonologists consider surgery and SABR to be comparable treatments in patients with early-stage NSCLC [22]. As such, distinguishing commonly observed benign fibrosis from early tumor recurrence will become increasingly important. Although radiological features suggestive of a local recurrence have been identified [23], both radiologists and radiation oncologists were less proficient in identifying recurrences, than by using image texture analysis (so-called radiomics) [24]. This suggests that image texture analysis may have a growing role in evaluating post-SABR radiology. ESMO guidelines recommend performing both a new ^18^FDG-PET scan and a biopsy of the suspected recurrence [1], as reports of benign lesions being excised after SABR are not uncommon [17, 25, 26]. The four patients in our series who underwent surgery without a biopsy reflects, in part, the fact that they were treated before the ESMO guidelines were published.

A number of key limitations of our study must be acknowledged. This study may not give a true incidence of surgical salvage for local failures after SABR as a large proportion of patients treated at both our centers were referred from the rest of the Netherlands. Consequently, some patients may have been operated upon elsewhere without our knowledge. Furthermore, only a minority of patients who undergo SABR are in fact medically operable, and all our patients represent cases of peripheral lung tumors, where central hilar and vascular structures did not receive significant doses of radiation. Consequently, the risks of post-operative complications could in fact be higher in a typical SABR population. We would suggest referral to expert surgical centers for such cases, until such time that broader experience becomes available.

In conclusion, following SABR for a peripheral NSCLC, radiological follow-up can allow for the timely detection and surgical excision of isolated local failures. Additional data to define optimal surgical approaches for such patients is awaited, as well as data in patients with more centrally located tumors.

## Conclusion

We report our experience with nine surgical procedures for local recurrences post-SABR. Our analysis revealed two grade IIIa complications and a 30-day mortality of 0 %. These results suggest that salvage surgery can be safely performed after SABR.
